# Association between probiotics and bronchopulmonary dysplasia in preterm infants

**DOI:** 10.1038/s41598-021-96489-z

**Published:** 2021-08-23

**Authors:** Yangming Qu, Shijie Guo, Ying Liu, Guohua Wang, Hui Wu

**Affiliations:** Department of Neonatology, The First Hospital of Jilin University, 1 Xinmin Street, Changchun, 130021 Jilin China

**Keywords:** Diseases, Health care

## Abstract

Bronchopulmonary dysplasia is a chronic pulmonary disease with a high incidence in premature infants, and there is still no effective treatment. The purpose of our study was to analyze the association between the use of probiotics and BPD in premature infants. We retrospectively collected clinical data of infants with gestational age < 32 weeks admitted to the NICU of The First Hospital of Jilin University from January 1, 2019 to March 31, 2020. Demographic and clinicopathological data of the inclusion population were collected. The outcome was the incidence of BPD or death. The χ^2^ tests was used to compare the categorical variables. The t test and non-parametric Wilcoxon rank-sum test were used to compare the continuous data. Univariate and multivariate logistic regression were used to analyze the association between probiotics and BPD. A total of 318 newborns met the inclusion criteria, of which 94 received probiotics and 224 received no probiotics. There were 16 deaths and 115 newborns with BPD in the included population. The results of univariate analysis showed differences in the maternal diabetes, the proportion of systemic antibiotics given to mother within 24 h before birth, the receiving rate of invasive mechanical ventilation, the prevalence of BPD/death, PDA, RDS and Ivh between newborns with and without probiotics (p < 0.05); The results of unadjusted univariate logistic regression model showed that probiotic (OR 0.034, 95% CI 0.012–0.096) was the factor affecting BPD in preterm infants (p < 0.05). Multivariate logistic regression result (OR 0.037, 95% CI 0.013–0.105) was consistent with univariate analysis (P < 0.001). Probiotics are associated with a reduced risk of BPD in preterm infants < 32 weeks of age. More prospective studies with large samples are still needed.

## Introduction

Bronchopulmonary dysplasia (BPD) is a chronic lung disease in premature infants, with a prevalence of 60–90% in extremely preterm infants (22–25 weeks gestation)^1–3^. Its long-term effects involve multiple organ systems such as pulmonary dysfunction and neurodysplasia^4,5^. Over the past few decades, the pathogenesis of BPD has evolved from pulmonary lesions secondary to mechanical ventilation and elevated oxygen concentrations to a multifactorial disease involving prenatal and postnatal factors^6^. Premature infants with decreased alveolarization and pulmonary vascular development are prone to BPD in the case of antenatal complications of chorioamnionitis^2,7,8^. In addition, clinical factors such as gestational age (GA), birth weight, gender, patent ductus arteriosus (PDA), the need for oxygen administration or mechanical ventilation, postnatal infections, and necrotizing enterocolonitis(NEC) have also been extensively studied as influencing factors of BPD^9–12^. The results of the meta-analysis showed that only Vitamin A and dexamethasone were effective in reducing the risk of BPD in preterm infants^13,14^. However, dexamethasone has limited use in preterm infants due to its side effects, and the treatment effect of vitamin A on BPD is weak^14,15^. Recently, regenerative medicine has also been suggested as a promising treatment for BPD. But more research is still needed, because the understanding of stem cell function is not complete^16^. Therefore, there is still a lack of effective treatment for BPD, and early prevention is very important. Probiotics are living microorganisms that can provide health benefits to their hosts when supplemented in appropriate doses. Although probiotics have been shown to reduce the risk of multiple diseases in preterm infants, no studies have examined the role of probiotics on BPD as a primary outcome^17^. The purpose of our study was to analyze the association between the use of probiotics and BPD in premature infants.

## Methods

### Study design

We retrospectively collected clinical data of patients admitted to the NICU of The First Hospital of Jilin University from January 1, 2019 to March 31, 2020. Infants with gestational age < 32 weeks who were admitted to the NICU within 24 h after birth were included in our study. Infants with severe congenital chest deformities, congenital heart disease, chromosomal abnormalities, or death before 36 weeks postmenstrual (PMA) were excluded. The Ethics Committee of the first hospital of Jilin University approved the study. Parental Informed consent was waived by the Ethics Committee of the first hospital of Jilin University as all data of our study are deidentified. All methods were performed in accordance with the relevant guidelines and regulations.

### Primary study exposure and covariates

The primary exposure in this study was probiotics. The probiotics used in our NICU are Clostridium butyricum Powder (LIVE). [trade name: Qingdao Donghai Pharmaceutical Co. Ltd., 0.5 g/bag]. Probiotics were mainly used in newborns with feeding intolerance, jaundice, or extremely premature delivery (once a day, one bag at a time). In our study, probiotics were defined as the use of probiotics > 4 days.

Covariates included demographic and clinicopathological characteristics of the study population. Demographic characteristics included gender, gestational age, PMA at the time of admission for the cohort, birth weight, apgar scores, type of feeding, transfer information, maternal age, type of delivery, number of births, prenatal medication, maternal diabetes and maternal hypertension. Clinicopathological characteristics included the use of respiratory support, probiotics, pulmonary surfactant and caffeine, and the incidence of patent ductus arteriosus (PDA), respiratory distress syndrome (RDS), retinopathy (ROP), necrotizing enterocolitis (NEC) and intraventricular Hemorrhage (IVH). Respiratory support included the use of oxygen on admission, non-invasive mechanical ventilation on admission, invasive mechanical ventilation on admission and duration of mechanical ventilation after admission. Diagnosis of PDA and RDS was made clinically^18^; ROP and IVH were diagnosed by an expert through examination of retinas of infants using indirect ophthalmoscope and cranial ultrasonographic scan^18,19^; NEC was defined as Bell classification II or greater^20^.

### Outcomes

The study outcome was the presence of BPD or death. BPD was defined as patients on any respiratory support at 36 weeks PMA. We further stratify the patients into the various Grades of BPD as described by Jensen et al.^21^ in the 2019 NRN criteria: no BPD, no support; grade 1 BPD, nasal cannula < 2 L/min; grade 2 BPD, nasal cannula > 2 L/min or non-invasive positive airway pressure; and grade 3 BPD, invasive mechanical ventilation.

### Statistical analysis

All statistical analyses were performed using SPSS statistical software, version 24.0 (IBM Corp, Armonk, NY). The normally distributed data were described as ${\overline{\text{x}}}$ ± S (mean ± standard deviation), the non-normally distributed data were described as median and quartile range, and the categorical variables were described as mummer and frequency. The χ^2^ tests was used to compare the categorical variables. The t test and non-parametric Wilcoxon rank-sum test were used to compare the continuous data. Univariate and multivariate logistic regression were used to analyze the association between probiotics and BPD.

### Ethics approval

The Ethics Committee of the first hospital of Jilin University approved the study.

### Consent to participate

Parental consent was waived as all data of our study are deidentified.

### Consent for publication

All co-authors agree to publish this manuscript.

## Results

### Demographic characteristics of the study population

A total of 318 newborns met the inclusion criteria, of which 94 received probiotics and 224 received no probiotics. There were 16 deaths (1 died of multiple organ failure, 4 died of neonatal sepsis, 1 died of heart failure, 3 died of septic shock, 1 died of necrotizing enterocolitis, 4 died of pulmonary hemorrhage < 14 days after birth, and 2 died of respiratory distress syndrome < 14 days after birth) and 115 newborns with BPD (38 patients had Grade 1 BPD, 26 had Grade 2 BPD, and 51 had Grade 3 BPD) in the included population. There are significant differences in the maternal diabetes, and the proportion of systemic antibiotics given to mother within 24 h before birth between newborns with and without probiotics (*p* < 0.05). The demographic characteristics of the study population were shown in Table 1.

**Table 1:** Demographic characteristics of the study population.

	Without probiotics (N = 224)	With probiotics (N = 94)	χ^2^/t	p
**Gender**			0.018	0.893
Male	121 (54%)	50 (53%)		
Female	103 (46%)	44 (47%)		
Gestational age(week)	29.9 ± 2.2	29.5 ± 2.0	1.366	0.173
Birth weight	1485.4 ± 422.1	1407.0 ± 373.3	1.563	0.119
**Apgar1**			0.060	0.807
≥ 7	132 (59%)	54 (57%)		
< 7	92 (41%)	40 (43%)		
**Apgar5**			0.191	0.662
≥ 7	144 (64%)	58 (62%)		
< 7	80 (36%)	36 (38%)		
**Type of feeding**			0.001	0.974
Breast milk	133 (59%)	56 (60%)		
Others	91 (41%)	38 (40%)		
**Maternal age (year)**			0.323	0.570
≤ 30	90 (40%)	41 (44%)		
> 30	134 (60%)	53 (56%)		
**Premature rupture of membranes**			2.523	0.112
No	149 (67%)	71 (76%)		
Yes	75 (34%)	23 (24%)		
**Type of delivery**			1.403	0.236
Vaginal	114 (51%)	41 (44%)		
Cesarean	110 (49%)	53 (56%)		
Number of births			0.022	0.881
1	165 (74%)	70 (75%)		
≥ 2	59 (26%)	24 (25%)		
**Receive antenatal corticosteroid**			3.587	0.058
No	122 (55%)	62 (66%)		
Yes	102 (45%)	32 (34%)		
**Receive antenatal MgSO**_**4**_			0.167	0.683
No	189 (84%)	81 (86%)		
Yes	35 (16%)	13 (14%)		
**Systemic antibiotics given to mother within 24 h before birth**			4.471	0.034
No	178 (80%)	84 (89%)		
Yes	46 (20%)	10 (11%)		
**Maternal diabetes**			4.277	0.039
No	185 (83%)	68 (72%)		
Yes	39 (17%)	26 (28%)		
**Maternal hypertension**			0.024	0.877
No	177 (79%)	75 (80%)		
Yes	47 (21%)	19 (20%)		
**Transfer**			0.213	0.645
No	53	20		
Yes	171	74		
**PMA at the time of admission for the cohort**	31.2 ± 2.7	30.8 ± 2.1	1.772	0.157

### Clinicopathological characteristics of study subjects

The clinicopathological characteristics of study subjects were shown in Table 2. The receiving rate of invasive mechanical ventilation was significantly different in newborns with and without probiotics (p < 0.05); In addition, there were also significant differences in the prevalence of BPD/death, PDA, RDS and Ivh between the two groups (p < 0.05).

**Table 2:** Clinicopathological characteristics of study subjects.

	Without probiotics (N = 224)	With probiotics (N = 94)	χ^2^/Z	p
**Oxygen administration**			2.623	0.105
No	78 (35%)	24 (26%)		
Yes	146 (65%)	70 (74%)		
**Noninvasive mechanical ventilation**			0.173	0.677
No	50 (22%)	19 (20%)		
Yes	174 (78%)	75 (80%)		
**Invasive mechanical ventilation**			11.880	0.001
No	140 (63%)	39 (42%)		
Yes	84 (37%)	55 (59%)		
Duration of mechanical ventilation after admission (days)	8 (3–14)	8.5 (3–18)	0.375	0.708
**BPD or death**			75.168	< 0.001
No	97 (43%)	90 (96%)		
Yes	127 (56%)	4 (4%)		
**PDA**			6.363	0.012
No	66 (30%)	15 (16%)		
Yes	158 (70%)	79 (84%)		
**RDS**			7.183	0.007
No	113 (50%)	32 (34%)		
Yes	111 (50%)	62 (66%)		
**Ivh**			9.871	0.002
No	129 (58%)	36 (38%)		
Yes	95 (42%)	58 (62%)		
**ROP**			1.813	0.178
No	64 (29%)	20 (21%)		
Yes	160 (71%)	74 (78%)		
**NEC**			2.615	0.106
No	221 (99%)	90 (96%)		
Yes	3 (1%)	4 (4%)		
**Receive caffeine**			0.218	0.641
No	144 (64%)	63 (67%)		
Yes	80 (36%)	31 (33%)		
**Pulmonary surfactant**			2.703	0.100
No	138 (62%)	67 (71%)		
Yes	86 (38%)	27 (29%)		

### Univariate and multivariate logistic regression analyses for the association between probiotics and BPD/death

With BPD as the dependent variable, the probiotics classification as the only covariate, the OR applied by unadjusted univariate logistic regression model was 0.034 (0.012–0.096) for the use of probiotics (p < 0.001). After adjustment for the proportion of systemic antibiotics given to mother within 24 h before birth, the receiving rate of invasive mechanical ventilation on admission, the prevalence of PDA, RDS and Ivh, the adjusted odds ratio was 0.037 (0.013–0.105) for the use of probiotics (p < 0.001) (Table 3).

**Table 3:** Univariate and multivariate logistic regression analyses for the association between probiotics and BPD/death.

	β	p	OR (95% CI)
Probiotics (yes vs no)			
Univariate analysis	− 3.383	< 0.001	0.034 (0.012–0.096)
Adjust model^a^	− 3.295	< 0.001	0.037 (0.013–0.105)

^a^Adjusted for baseline maternal diabetes, the proportion of systemic antibiotics given to mother within 24 h before birth, the receiving rate of invasive mechanical ventilation, the prevalence of PDA, RDS and Ivh.

## Discussions

In our study, univariate logistic regression analysis showed a protective effect of probiotics on BPD in preterm infants with gestational age less than 32 weeks. The adjusted multivariate logistic regression results were consistent with the univariate logistic regression, and the influence of probiotics was slightly diminished.

Probiotics are supplements of living micro-organisms that colonize the gut. Proper probiotics can confer a benefit on the host by regulating local and systemic immunity and increasing anti-inflammatory cytokines^22^. Several meta-analyses of randomized controlled studies have shown that the probiotics supplementation can reduce neonatal mortality, necrotizing enterocolitis, and late-onset sepsis, as well as the time to achieve full enteral feeding in preterm infants^23–25^. Inflammatory events such as necrotizing enterocolitis and late-onset sepsis are also important influencing factors of BPD, so the preventive effect of probiotics on BPD is also worth expecting. Recently, a meta-analysis reported that available evidence could not support any significant effect of probiotics on reducing the incidence of BPD^17^. However, the authors also stressed that their results should be interpreted with caution, as the included studies showed methodological differences in terms of inclusion criteria, timing, dosage and probiotic formulations used. Moreover, BPD was not a primary outcome in any study, and the number of probiotics studies that included BPD as a secondary outcome was relatively small. In addition, few studies have focused on preterm infants < 32 weeks of gestational age, and none of the included studies specifically targeted the most vulnerable group (infants < 28 weeks of gestational age) of BPD. Therefore, the effect of probiotics on BPD in premature infants is still unclear. Our study is the first to use the effect of probiotics on BPD as the primary outcome. We found that the use of probiotic was associated with reduced BPD in preterm infants < 32 weeks of age.

The protective effect of probiotics on BPD may have several hypothesized mechanisms : (1) Premature infants have immature immune systems that cannot balance the pro-inflammatory responses, leading to a decrease in the number of regulatory T cells (Tregs) that constitute anti-inflammatory lymphocyte subsets and a higher proportion of activated pro-inflammatory T cells, which is an important cause of BPD. Probiotics appear to improve Treg production, expansion, and activity while reducing the activation of proinflammatory lymphocyte subsets^26,27^; (2) Probiotics can reduce the occurrence of BPD by regulating intestinal flora^28^; (3) In the NEC experiment, the combined effect of hyperoxia and suboptimal nutrition had an adverse effect on the level of lung vascular endothelial growth factor (PVEGF). Probiotics can improve the nutritional status of infants, help to improve lung vasculogenesis and prevent BPD^25,26,29^; (4) In addition, the performance of probiotics also help to reduce the incidence of BPD^30^.

There are some limitations in our study: (1) our study is a single-center retrospective study in which the study population was grouped according to probiotics use. Since probiotics were used prophylactically in most cases, parental refusal to use probiotics may affect the results of this study. (2) Although our study showed that the use of probiotics had a protective effect on BPD, it did not analyze the effects of different types of corticosteroid or strains, the optimal dose and duration of use were not analyzed. (3) BPD is a disease with many risk factors, and factors not included may affect the results of this study.

## Conclusions

Probiotics are associated with a reduced risk of BPD in preterm infants < 32 weeks of age. More prospective studies with large samples are still needed.

## Data Availability

The datasets used and/or analysed during the current study are available from the corresponding author on reasonable request.
